# Supplementary material for: Proliferative polyploid cells give rise to tumors via ploidy reduction
Source: Nat Commun. 2021 Jan 28;12:646. doi: 10.1038/s41467-021-20916-y (PMC7843634; doi:10.1038/s41467-021-20916-y)
Supplement: Supplementary file 1 — Supplementary Information [file 41467_2021_20916_MOESM1_ESM.pdf]

Proliferative polyploid cells give rise to tumors via ploidy reduction

Tomonori Matsumoto[1]*, Leslie Wakefield[1], Alexander Peters[1], Myron Peto[2], Paul Spellman[2], and Markus Grompe[1]*

Supplementary Figures 1-10

Supplementary Fig. 1

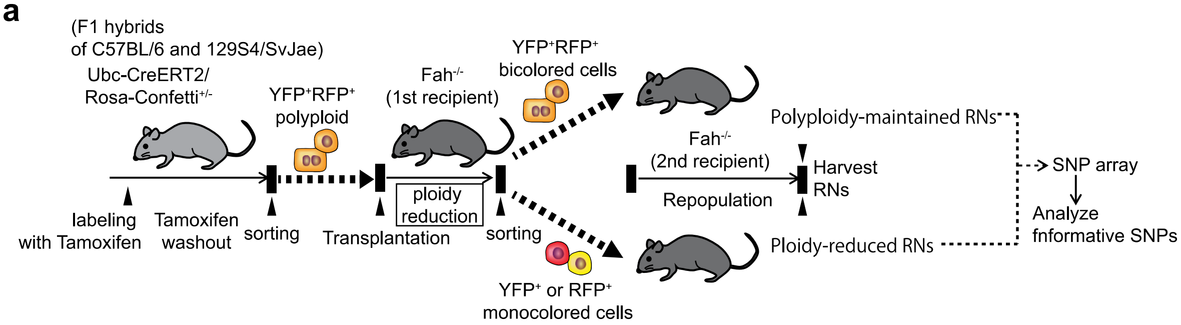

c

Bicolored RN

Sample	Chromosome aberrations
#1	1(129)+, 4(129)-, 11(C57)-, 16(129)-
#2	10(129)-, 12(C57>129)*
#3	4(129)-, 10(C57)-
#4	4(C57>129)*
#5	16(129)+
#6 - #17	No allele imlabances

Monocolored RN

Sample	Chromosome aberrations
#18	1(129)+, 2(C57)-, 3(C57>129)*, 4(C57)-, 5(C57>129)*, 6(129)-, 7(C57>129)*, 9(129)-, 10(129)+, 11(C57)-, 13(C57>129)*, 14(129)-, 15(129)+, 17(129>C57)*, 18(129)+, 19(C57)+
#19	3(129)+, 9(129>C57)*, 16(C57)-
#20	1(129)+
#21	1(129)+
#22	9(C57)+
#23	12(129)-
#24	12(C57)-
#25	13(C57)-
#26	14(C57)-
#27	19(129)+
#28	19(C57>129)*
#29 - #39	No allele imlabances

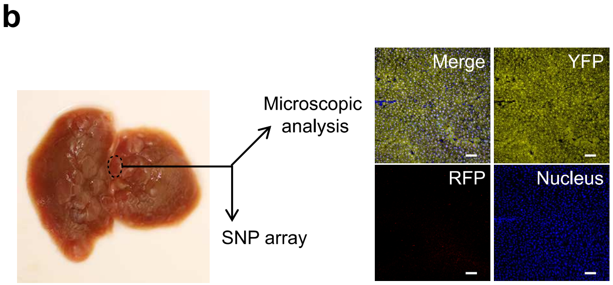

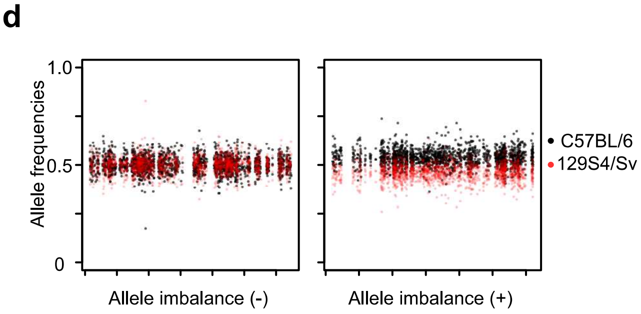

Supplementary Fig. 1. Detecting chromosome aberrations in regenerative nodules. (a) Experimental scheme to analyze polyploid-maintained and ploidy-reduced RNs. Only one of four fluorescent reporters (GFP, YFP, RFP, and CFP) is stochastically expressed from each Rosa-Confetti allele after cre recombination. In heterozygous Rosa-Confetti mice, ploidy reduction of multicolored polyploid hepatocytes can be traced as loss of a reporter expression. SNP informative heterozygous Ubc-CreERT2/Rosa-Confetti$^{+/-}$ mice were labeled with administration of tamoxifen, and YFP^{+}RFP^{+} bicolored polyploid cells were sorted by fluorescence-activated cell sorting (FACS) after a tamoxifen washout period of at least 3 weeks. Sorted cells were transplanted into first Fah$^{-/-}$ recipient mice, and bicolored donor cells gave rise to monocolored daughter cells via ploidy reduction during repopulating the recipient livers (see Fig. 1c). YFP^{+}RFP^{+} bicolored cells and either YFP^{+} or RFP^{+} monocolored cells were separately sorted from the recipient livers and transplanted into secondary recipient mice. RNs in the secondary recipients were subjected to analysis. (b) Representative liver and RNs of secondary recipient mice. One of RNs is indicated by a dotted circle. Harvested RNs were split into two pieces, and one of them was analyzed by microscopy after fixation to confirm clonal reporter expressions in RNs. Representative microscopic image of a monocolored RN is shown. The other piece was subjected to DNA extraction followed by SNP array analysis. Scale bars = 100 μm. (c) Overview of SNP array results. C57 and 129 in parentheses indicate alterations of C57BL/6- and 129S4/SvJae-derived alleles, respectively. Asterisks are allele imbalance events, whose copy number alterations didn't reach significance. (d) Representative data of high-density SNP array analysis. Results of the same chromosomes as those shown in Fig. 1d are shown.

Supplementary Fig. 2

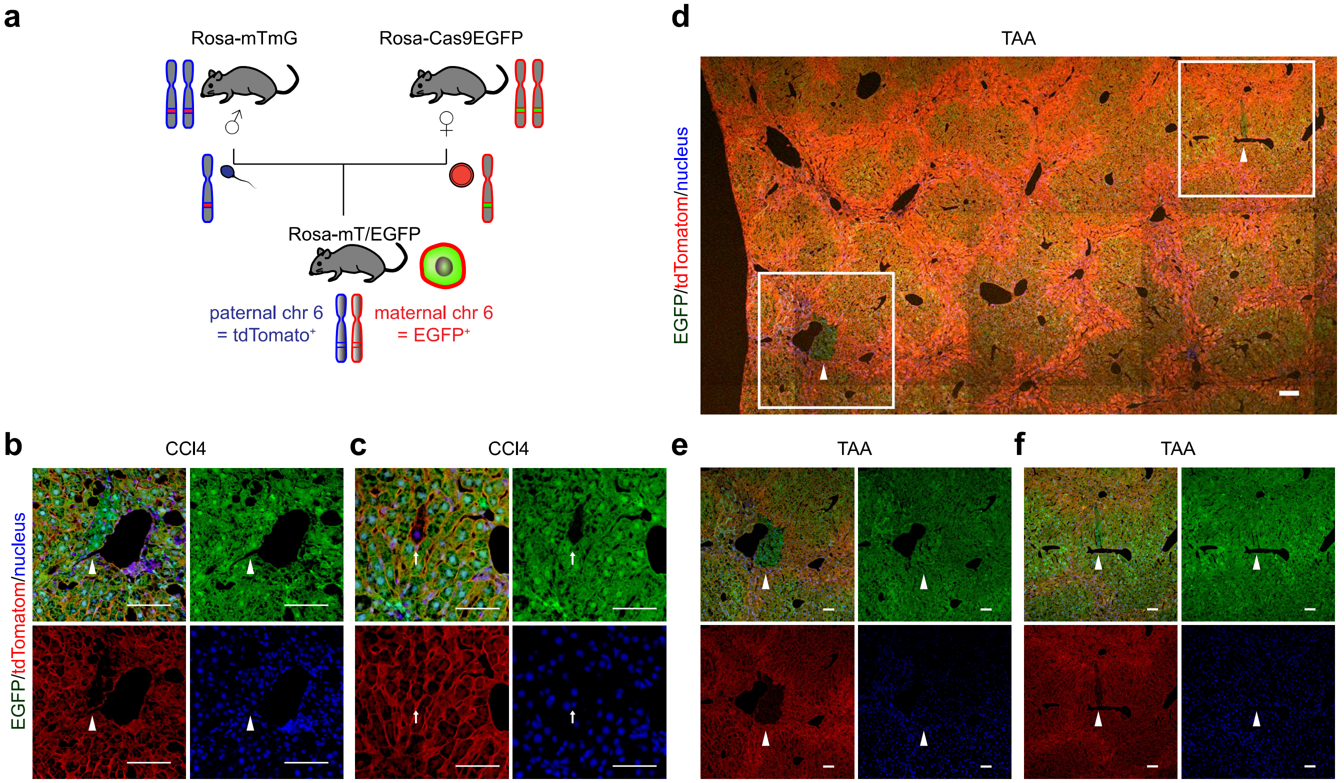

Supplementary Fig. 2. Detecting loss of heterozygosity in the regenerating liver. (a) Scheme for the detection of loss of heterozygosity. Blue and red chromosomes indicate paternal and maternal chromosomes, respectively. Both reporters are located in the Rosa locus and therefore loss of heterozygosity of chromosome 6 can be visualized by the presence of only one reporter. (b, c, d, e, f) Representative microscopic images of the livers of Rosa-mT/EGFP mice after CCl4 (b, c) and TAA (d, e, f) injuries. The livers were harvested at about 2 months and 3 months after the initiation of injuries in CCl4 and TAA, respectively. Monocolored cells positive for only tdTomato or EGFP are indicated by arrows and arrowheads, respectively. (e) and (f) are the high-magnification images of indicated areas in (d). In (d), images were stitched to generate large composite images. Scale bars = 100 μm.

Supplementary Fig. 3

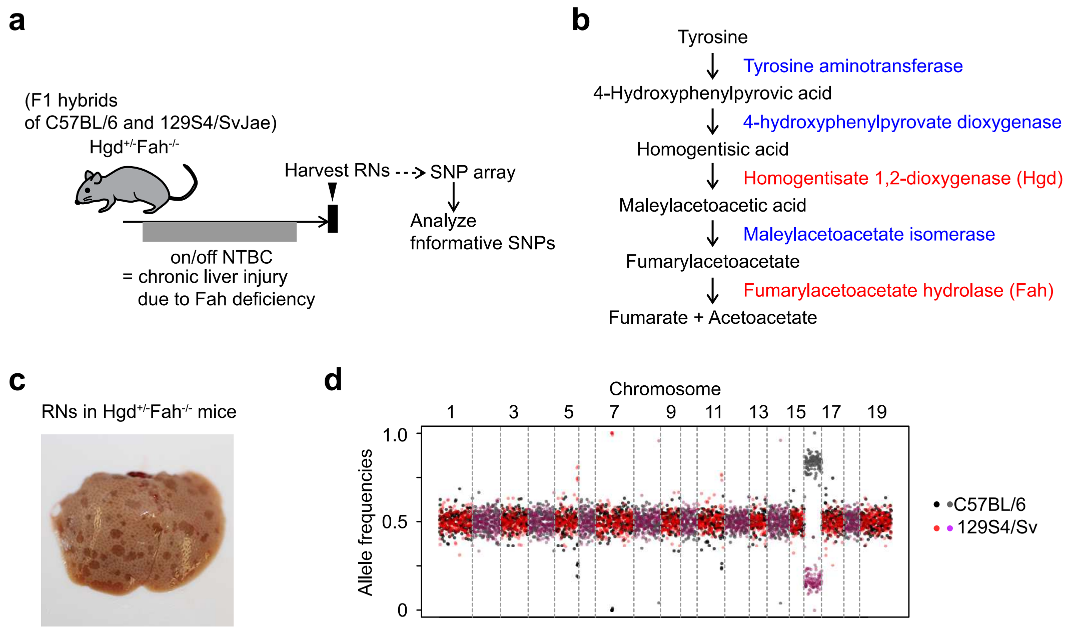

Supplementary Fig. 3. Adaptation by chromosome aberrations in the regenerating liver. (a) Experimental scheme to analyze RNs in Hgd$^{+/-}$Fah$^{-/-}$ mice. Mice were harvested after 5-7 months of liver injury. (b) Diagram of the tyrosine catabolic pathway. Loss of Fah leads to accumulation of fumarylacetoacetate and toxic metabolites. Fah deficiency is ameliorated by loss of Hgd. (c) Representative liver containing RNs in Hgd$^{+/-}$Fah$^{-/-}$ mice. (d) Representative SNP array data indicating allele imbalance in chromosome 16 encoding *Hgd* gene. 129S4/SvJae- and C57BL/6-derived chromosome 16 are proficient and deficient for *Hgd* gene, respectively. Note that 129S4/SvJae-derived chromosome 16 that is proficient for *Hgd* is lost, which results in hepatocyte adaptation in Fah deficient livers.

Supplementary Fig. 4

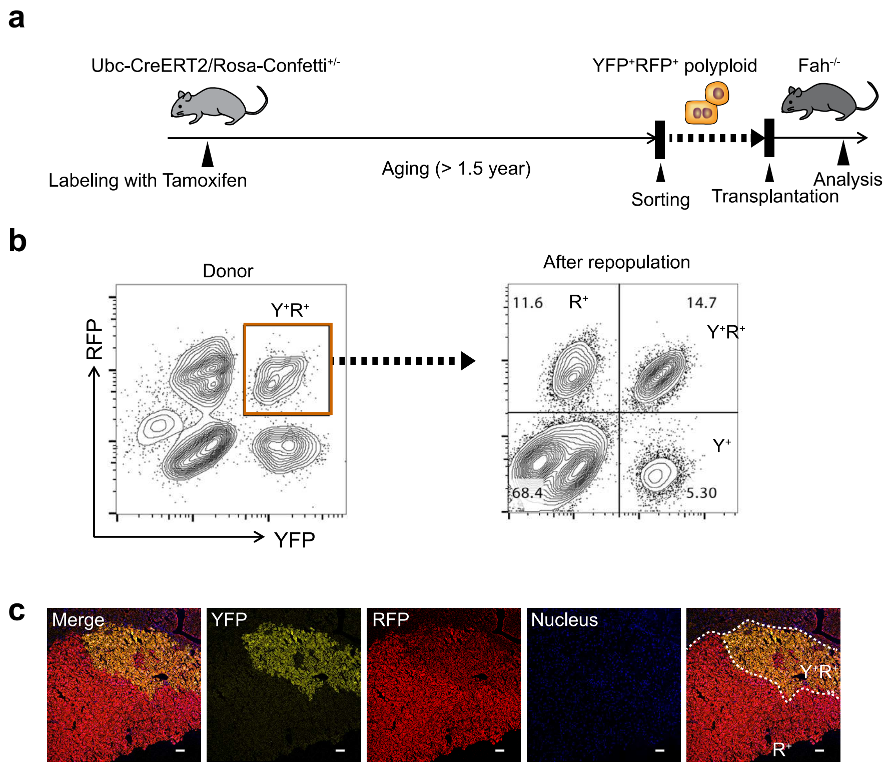

Supplementary Figure 4. Ploidy reduction in aged polyploid hepatocytes. (a) Experimental scheme of transplantation of aged polypoid hepatocytes into $Fah^{-/-}$ mice. Bicolored polyploids from aged mice (22-month-old) labelled at age 3 months were transplanted into $Fah^{-/-}$ recipients. (b) FACS plots before and after transplantation. (c) Representative microscopic images of recipient liver transplanted with aged polyploid hepatocytes. Each clonally repopulated area is indicated by dashed lines. Scale bars = 100 μm. Y^{+}, $EYFP^{+}$; R^{+}, RFP^{+}.

Supplementary Fig. 5

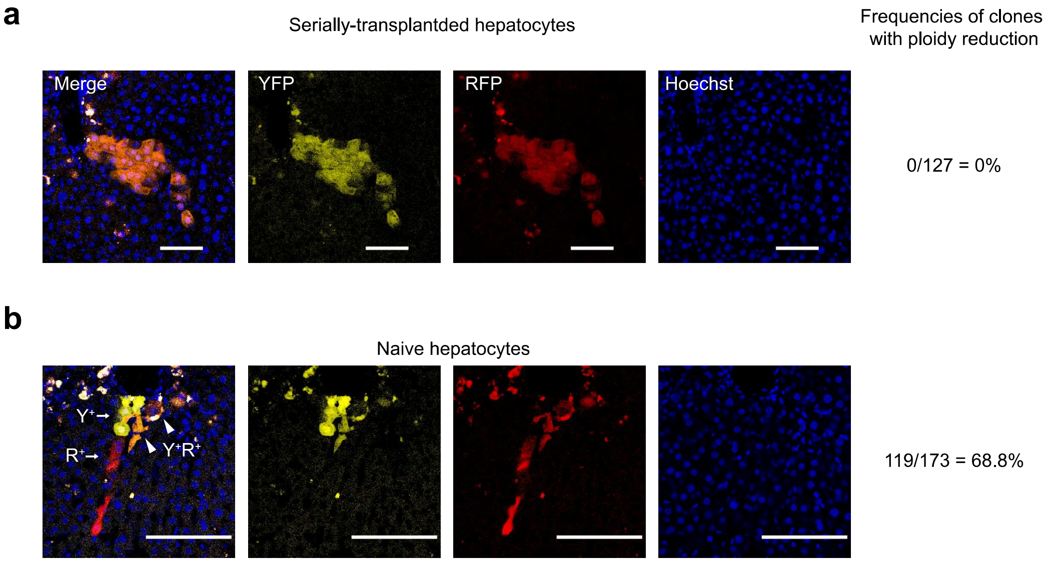

Supplementary Figure 5. Ploidy reduction during CCl4-induced liver injury. (a, b) Representative microscopic images of clones derived from transplanted bicolored polyploid hepatocytes after CCl4-induced liver injury. YFP^+RFP^+ polyploid hepatocytes heterozygous for Rosa-Confetti were sorted from serially-transplanted livers (a) or naïve liver (b), and were subjected to CCl4-induced liver injury after transplantation into wild-type mice. Briefly, after 2 weeks of engraftment period, the livers were injured with CCl4 for 6 weeks, and harvested for microscopic analysis. Tissues were analyzed by 3D imaging of 250-μm thick slices, and representative Z images are shown. Serially-transplanted hepatocytes did not give rise to monocolored cells (a), whereas naïve hepatocytes sometimes generated monocolored cells indicating ploidy reduction (b). Frequencies of clones with ploidy reduction among those analyzed are also indicated. Scale bars = 100 μm. Y^+, $EYFP^+$; R^+, RFP^+.

Supplementary Fig. 6

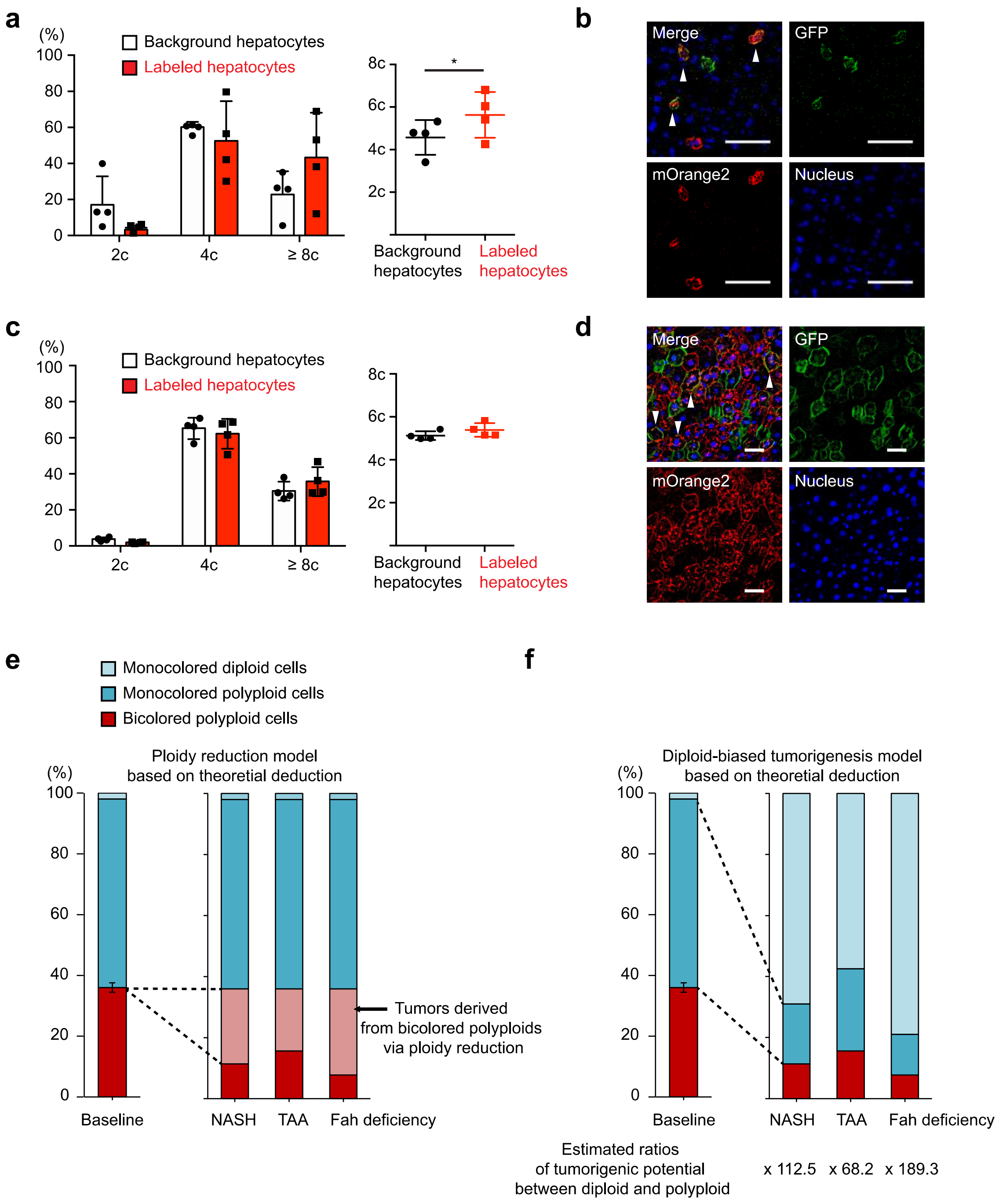

Supplementary Figure 6. Tracing of polyploid-derived hepatocarcinogenesis in Rosa-RGBow$^{+/-}$ mice. (a, c) Ploidies of labeled and background hepatocytes in Rosa-RGBow$^{+/-}$ mice that received hydrodynamic injection of plasmids (a) and those infected with AAV8-Ttr-Cre (c). Difference of average ploidy between labeled and background hepatocytes was statistically analyzed by two-sided paired *t*-test. Asterisk indicates significant difference (p = 0.011). Error bars indicate mean ± SD. (n = 4 biologically independent mice). (b, d) Representative microscopic images of the Rosa-RGBow$^{+/-}$ liver after hydrodynamic injection (b) and that infected with AAV8-Ttr-Cre (d). mKate2-positive cells are not shown because they are quite rare. Some representative EGFP^{+} and mOrange^{+} double-positive cells are indicated by arrowheads. Scale bars = 50 μm. (e, f) Two models to explain decreased frequencies of bicolored tumors in chronically injured livers. (e) Ploidy reduction model. If ploidy reduction occurs during tumorigenesis derived from bicolored polyploid hepatocytes, the frequency of bicolored tumors among labeled tumors decreases. (f) Diploid-biased tumorigenesis model. The decreased frequencies of bicolored tumors can also be explained if monocolored diploid hepatocytes have higher tumorigenic potential than labeled polyploid hepatocytes. Tumorigenic potential of diploids compared to polyploids were estimated under a condition where no ploidy reduction occur during tumorigenesis. Note that diploids must have much higher tumorigenic potential than polyploids in this model because monocolored diploids account for only about 2% of labeled cells (see Supplementary Fig. 6c). Note that (e) and (f) are based on theoretical deduction using reporter expressions, not actual ploidy data of tumors because it is difficult to deduce tumor origins by ploidy status of established tumors due to frequent ploidy alterations (polyploidization and ploidy reduction) of hepatocytes during tumorigenesis. Labeling frequencies of the Rosa-RGBow$^{+/-}$ livers without liver injuries are shown in parallel as a baseline. Source data are provided as a Source Data file.

Supplementary Fig. 7

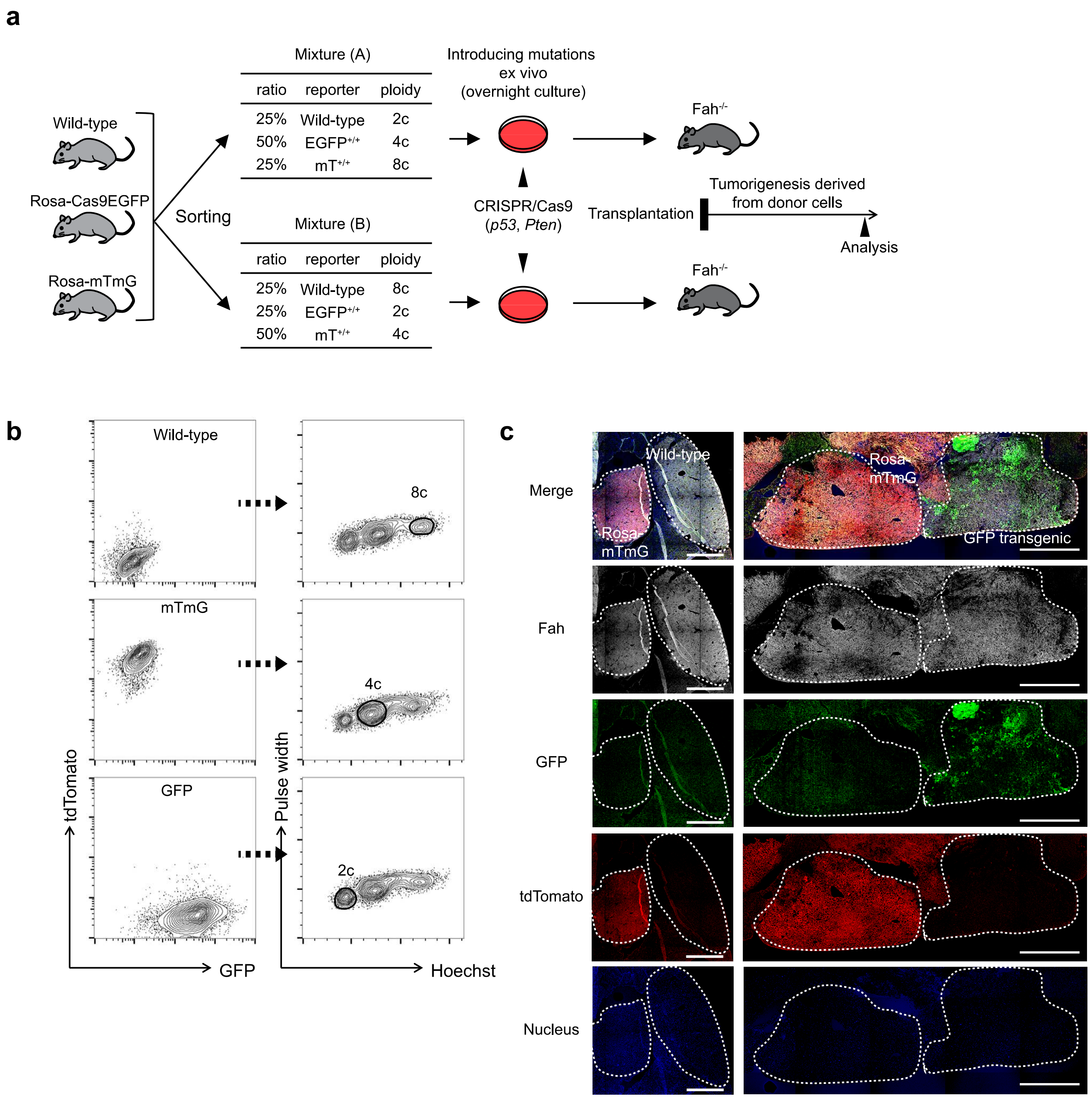

Supplementary Figure 7. Comparison of tumorigenic potential between diploid, tetraploid, and octaploid hepatocytes. (a) Experimental scheme to compare tumorigenic potential between diploid, tetraploid, and octaploid cells. Non-colored, $EGFP^{+/+}$, and $tdTomato^{+/+}$ cells were sorted from homozygous wild-type, GFP transgenic, and Rosa-mTmG mice, respectively. Sorted cells were mixed as indicated and transfected with CRISPR/Cas9 targeting for *p53* and *Pten* genes. Cells were transplanted into $Fah^{-/-}$ recipients after overnight culture ex vivo. Tumorigenic potential was calculated by adjusting numbers of tumors derived from each ploidy to input cell amount. Tumors were analyzed about 3-4 months after transplantation. (b) Representative FACS plots during sorting. (c) Representative microscopic images of tumors. To detect non-colored tumors, tissues were stained with Fah. Each tumor is indicated by dashed lines. Images were stitched to generate large composite images. Scale bars = 100 μm.

Supplementary Fig. 8

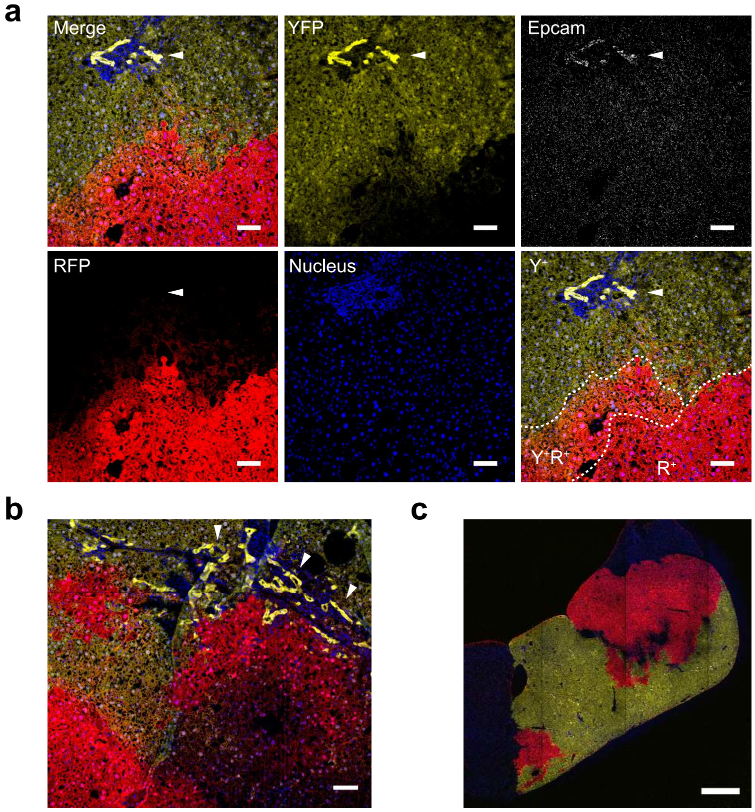

Supplementary Figure 8. Intratumoral ploidy reduction accompanied by cholangiocellular transdifferentiation. Another tumor different from that in Fig. 4h is shown. (a), (b), and (c) are different sections of the same tumor. Transdifferentiation into Epcam-positive cholangiocellular cells were indicated by arrow heads. Each clonal tumor area is indicated by dashed lines. Images were stitched to generate large composite images. Y^+, $EYFP^+$; R^+, RFP^+. Scale bars = 100 μm.

Supplementary Fig. 9

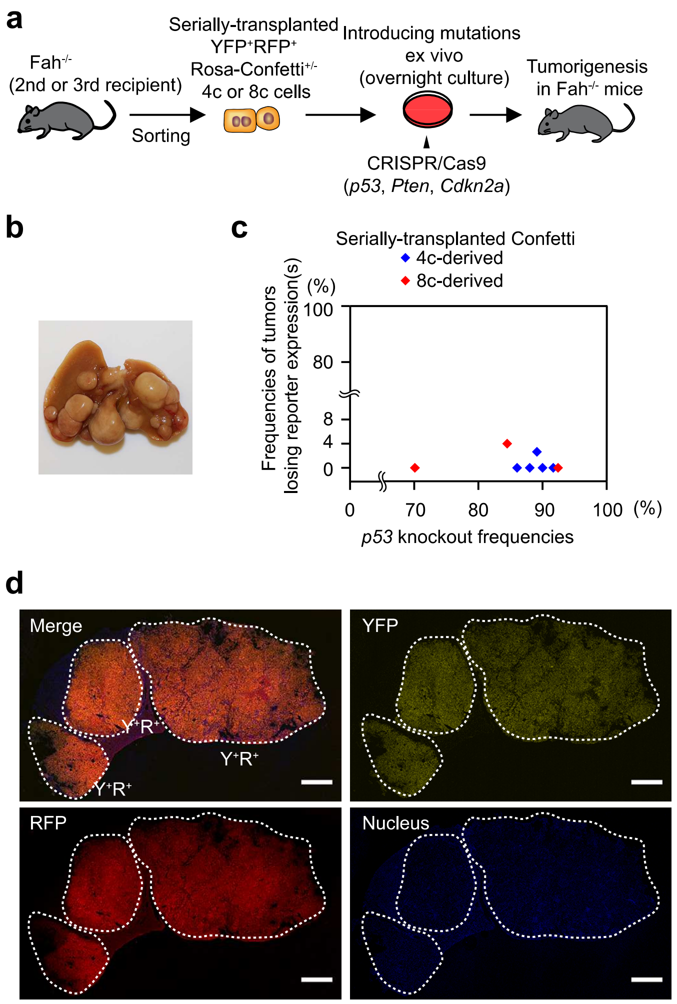

Supplementary Figure 9. Suppressed ploidy reduction during tumorigenesis derived from serially-transplanted polyploid hepatocytes. (a) Experimental scheme of tumorigenesis derived from serially-transplanted polyploid hepatocytes. YFP$^+$RFP$^+$ hepatocytes heterozygous for Rosa-Confetti were sorted from secondary or tertiary recipient Fah$^{-/-}$ mice. (b) Representative macroscopic images of the liver with tumors derived from serially-transplanted hepatocytes. (c) Frequencies of tumors that lost reporter expression(s) and their correlation with *p53* knockout frequencies in origin cells. Tumors were analyzed about 4 months after transplantation, and 23.5 tumors were analyzed in each mouse on average. Knockout efficiencies by CRISPR/Cas9 in origin cells were analyzed by TIDE. Note marked contrast with Fig. 4e. (d) Representative microscopic images of the liver with tumors derived from serially-transplanted hepatocytes. Images were stitched to generate large composite images. Y$^+$, EYFP$^+$; R$^+$, RFP$^+$. Scale bars = 100 μm. Source data are provided as a Source Data file.

Supplementary Fig. 10

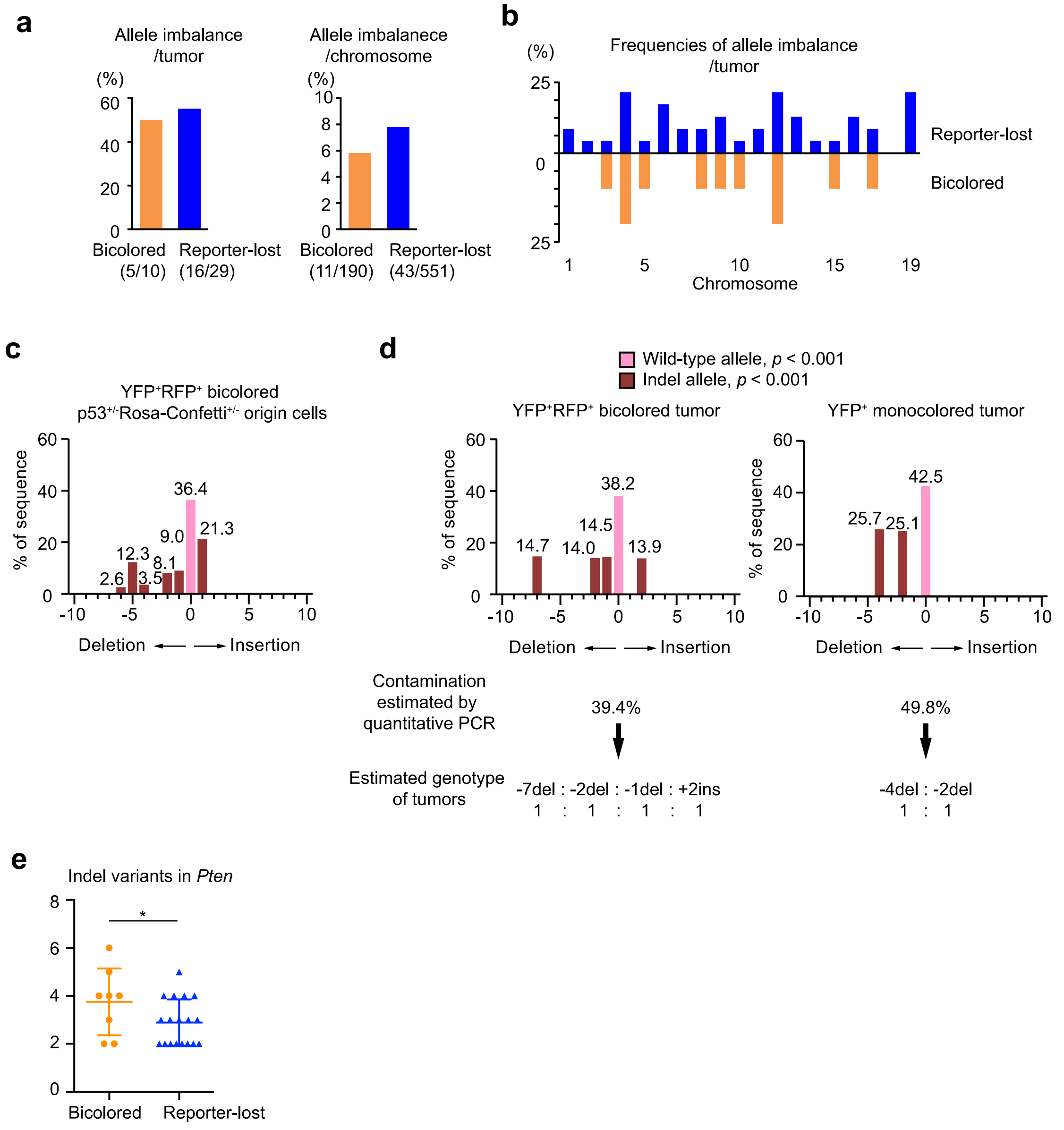

Supplementary Figure 10. Allele imbalances and concentrated oncogenic chromosomes in ploidy-reduced tumors. (a) Frequencies of AIs in tumors detected by SNP array. Values in parentheses indicate numbers of tumors or chromosomes with allele imbalances among those analyzed. Only autosomes were analyzed. (b) Frequencies of AIs in each chromosome. (c, d) Representative TIDE results of Pten gene in origin cells (c) and tumors (d). Estimation of contamination from non-tumorous recipient cells was performed using quantitative PCR for Fah gene. Note that wild-type alleles in tumors detected by TIDE were accounted for by contaminating DNAs derived from Fah$^{-/-}$ recipient cells. (e) Variation of Pten indels in tumors. Biologically independent tumors were analyzed. Asterisk indicates significant difference in a one-sided unpaired *t* test (p = 0.039). Error bars indicate mean ± SD (n = 8 in bicolored tumors, and n = 18 in reporter-lost tumors). Source data are provided as a Source Data file.
