# Supplementary material for: Proliferative polyploid cells give rise to tumors via ploidy reduction
Source: Nat Commun. 2021 Jan 28;12:646. doi: 10.1038/s41467-021-20916-y (PMC7843634; doi:10.1038/s41467-021-20916-y)
Supplement: Supplementary file 3 — Reporting Summary [file 41467_2021_20916_MOESM3_ESM.pdf]

nature research

Corresponding author(s): Tomonori Matsumoto
Markus Grompe

Last updated by author(s): Nov 21, 2020

Reporting Summary

Nature Research wishes to improve the reproducibility of the work that we publish. This form provides structure for consistency and transparency in reporting. For further information on Nature Research policies, see Authors & Referees and the Editorial Policy Checklist.

Statistics

For all statistical analyses, confirm that the following items are present in the figure legend, table legend, main text, or Methods section.

n/a	Confirmed	
☐	☒	The exact sample size (n) for each experimental group/condition, given as a discrete number and unit of measurement
☐	☒	A statement on whether measurements were taken from distinct samples or whether the same sample was measured repeatedly
☐	☒	The statistical test(s) used AND whether they are one- or two-sided<br>*Only common tests should be described solely by name; describe more complex techniques in the Methods section.*
☒	☐	A description of all covariates tested
☒	☐	A description of any assumptions or corrections, such as tests of normality and adjustment for multiple comparisons
☐	☒	A full description of the statistical parameters including central tendency (e.g. means) or other basic estimates (e.g. regression coefficient) AND variation (e.g. standard deviation) or associated estimates of uncertainty (e.g. confidence intervals)
☐	☒	For null hypothesis testing, the test statistic (e.g. F, t, r) with confidence intervals, effect sizes, degrees of freedom and P value noted<br>*Give P values as exact values whenever suitable.*
☒	☐	For Bayesian analysis, information on the choice of priors and Markov chain Monte Carlo settings
☒	☐	For hierarchical and complex designs, identification of the appropriate level for tests and full reporting of outcomes
☒	☐	Estimates of effect sizes (e.g. Cohen's d, Pearson's r), indicating how they were calculated

Our web collection on statistics for biologists contains articles on many of the points above.

Software and code

Policy information about availability of computer code

Data collection	Zeiss Zen software (ver. 2.3 Blue edition, Carl Zeiss AG)
Data analysis	R software (ver. 3.4.3, R Foundation for Statistical Computing): all codes used were based on commonly used formulas in R<br>Microsoft Excel 2013 (ver. 15.0, Microsoft)<br>Prism 7 (GraphPad Software, Inc.)<br>FlowJo (ver. 10.6.0, TreeStar)<br>GenomeStudio (ver. 2.0.5, Illumina)

For manuscripts utilizing custom algorithms or software that are central to the research but not yet described in published literature, software must be made available to editors/reviewers. We strongly encourage code deposition in a community repository (e.g. GitHub). See the Nature Research guidelines for submitting code & software for further information.

Data

Policy information about availability of data

All manuscripts must include a data availability statement. This statement should provide the following information, where applicable:
- Accession codes, unique identifiers, or web links for publicly available datasets
- A list of figures that have associated raw data
- A description of any restrictions on data availability

The authors declare that all data supporting the findings of this study are available within the article, its Extended Data, or from the corresponding author upon reasonable request.

Field-specific reporting

Please select the one below that is the best fit for your research. If you are not sure, read the appropriate sections before making your selection.

☒ Life sciences ☐ Behavioural & social sciences ☐ Ecological, evolutionary & environmental sciences

For a reference copy of the document with all sections, see nature.com/documents/nr-reporting-summary-flat.pdf

Life sciences study design

All studies must disclose on these points even when the disclosure is negative.

Sample size	No statistical tests were used to determine sample size. Number of sample was determined based on experimental approach, availability, and feasibility required to obtain definitive results. Specifically, we utilized sample sizes previously described in the literature of similar experiments.
Data exclusions	No data was excluded.
Replication	All repeatable experiments are repeated 3 or more times. In experiments to analyze mice comprehensively, mice were analysed in 3 or more batches. All experiments were successfully reproduced.
Randomization	Mice were randomly allocated into experimental groups.
Blinding	In SNP array analysis, both bicolored and reporter-lost samples were analyzed at the same time without information on their reporter expressions, and detected allele imbalances were referred to their sample information later.<br>For other experiments, blinding was not conducted during experiments because each experiment was performed by a single investigator and because collected data were quantitative and not influenced by investigator's bias.

Reporting for specific materials, systems and methods

We require information from authors about some types of materials, experimental systems and methods used in many studies. Here, indicate whether each material, system or method listed is relevant to your study. If you are not sure if a list item applies to your research, read the appropriate section before selecting a response.

Materials & experimental systems

n/a	Involved in the study	
☐	☒	Antibodies
☒	☐	Eukaryotic cell lines
☒	☐	Palaeontology
☐	☒	Animals and other organisms
☒	☐	Human research participants
☒	☐	Clinical data

Methods

n/a	Involved in the study	
☒	☐	ChIP-seq
☐	☒	Flow cytometry
☒	☐	MRI-based neuroimaging

Antibodies

Antibodies used	anti-Pericentrin (abcam, ab4448)<br>Alexa Fluor 647-conjugated anti-gamma Tubulin (abcam, ab191114)<br>rat anti-Epcam (Becton Dickenson, 552370)<br>rabbit anti-Fah (Overturf, K. et al. Nat Genet, 1996)<br>Alexa Fluor 647-conjugated anti-rat (Jackson ImmunoResearchm, 712-605-153)<br>Alexa Fluor 647-conjugated anti-rabbit (Jackson ImmunoResearch, 711-605-152)<br>Alexa Fluor 488-conjugated anti-rabbit (Jackson ImmunoResearch, 111-545-144)
Validation	All commercially available antibodies are validated by suppliers as follows.<br>https://www.abcam.com/pericentrin-antibody-centrosome-marker-ab4448.html<br>https://www.abcam.com/gamma-tubulin-antibody-tu-30-c-terminal-alexa-fluor-647-ab191114.html<br>https://www.bdbiosciences.com/us/applications/research/stem-cell-research/cancer-research/human/purified-rat-anti-mouse-cd326-g88/p/552370<br>https://www.jacksonimmuno.com/catalog/products/712-605-153<br>https://www.jacksonimmuno.com/catalog/products/711-605-152<br>https://www.jacksonimmuno.com/catalog/products/111-545-144<br><br>Rabbit anti-Fah is validated in the following paper and other researchers.<br>Overturf, K. et al. Nat Genet. 1996;12:266-73. doi: 10.1038/ng0396-266

Animals and other organisms

Policy information about studies involving animals; ARRIVE guidelines recommended for reporting animal research

Laboratory animals	Ubc-CreERT2, Rosa-Confetti, Rosa-RGBow, Rosa-mTomato/mGFP (Rosa-mTmG), and Rosa-Cas9EGFP were obtained from The Jackson Laboratory, and maintained on the C57BL/6 background. Trp53 mutant mice were obtained from Dr. Allan Bradley (Department of Medicine, University of Cambridge) and maintained on the 129S4/SvJae background. GFP transgenic mice were obtained from Dr. Masaru Okabe (Osaka University). Fah-/- mice on either the C57BL/6 or the 129S4/SvJae background were generated in our laboratory and reported previously (Grompe, M. et al. Genes Dev,1993). C57BL/6 wild-type mice were obtained from The Jackson Laboratory. 129S4/SvJae wild-type mice were obtained from Dr. Philippe M Soriano (Icahn School of Medicine at Mount Sinai). 5-12 week-old male and female Fah -/- mice were used as recipients. 6-20 week-old male and female mice were used for other experiments.
Wild animals	No wild animals were used in this study.
Field-collected samples	No field-collected samples were used in this study.
Ethics oversight	The Oregon Health & Science University Institutional Animal Care and Use Committee (Portland, OR) approved all animal experiments described.

Note that full information on the approval of the study protocol must also be provided in the manuscript.

Flow Cytometry

Plots

Confirm that:

☒ The axis labels state the marker and fluorochrome used (e.g. CD4-FITC).

☒ The axis scales are clearly visible. Include numbers along axes only for bottom left plot of group (a 'group' is an analysis of identical markers).

☒ All plots are contour plots with outliers or pseudocolor plots.

☒ A numerical value for number of cells or percentage (with statistics) is provided.

Methodology

Sample preparation	Primary hepatocytes were isolated by a two-step collagenase perfusion method46, and collected by sequential centrifugation at 50 g × 2 minutes.
Instrument	FACS was performed with a Cytopeia inFluxV-GS (Becton Dickenson, Franklin Lakes, NJ) at a flow rate of about 1,000 cells/sec. Flow cytometric analysis was performed with a BDLSRFortessa (Becton Dickenson), BD FACSymphony (Becton Dickenson), or Cytoflex S (Beckman Coulter Life Sciences, Indianapolis, IN).
Software	Data were processed using FlowJo software (TreeStar).
Cell population abundance	The abundance of cells was dependent on experiments. More than 1 million hepatocytes were typically analyzed, and more than 50 thousands of viable fluorophore-positive cells were typically analyzed.
Gating strategy	Endogenous fluorophore-negative and fluorophore-positive cells were used to establish gates for each fraction. Gates were drawn to collect cells expressing either fluorophore. See the provided examples for gates used.

☒ Tick this box to confirm that a figure exemplifying the gating strategy is provided in the Supplementary Information.
